# Forgiveness of INSTI-Containing Regimens at Drug Concentrations Simulating Variable Adherence *In Vitro*

**DOI:** 10.1128/aac.02038-21

**Published:** 2022-04-07

**Authors:** Rima K. Acosta, Michelle L. D’Antoni, Andrew Mulato, Stephen R. Yant, Tomas Cihlar, Kirsten L. White

**Affiliations:** a Gilead Sciences, Inc., Foster City, California, USA

**Keywords:** bictegravir, forgiveness, resistance

## Abstract

The integrase strand transfer inhibitor (INSTI)-based regimens bictegravir/emtricitabine/tenofovir alafenamide (BIC/FTC/TAF), dolutegravir (DTG)+FTC/TAF, DTG/lamivudine (3TC), and DTG/rilpivirine (RPV) are all approved for treatment of HIV-infected patients, with various limitations. Here, time to *in vitro* viral breakthrough (VB) and resistance barrier using simulated human drug exposures at either full or suboptimal treatment adherence to each regimen were compared. At drug concentrations corresponding to full adherence and 1 missed dose (*C*_min_ and *C*_min_−1), no VB occurred with any regimen. At *C*_min_−2, VB occurred only with DTG+3TC, with emergent resistance to both drugs. At *C*_min_−3, VB occurred with all regimens: 100% of DTG+3TC cultures had VB by day 12, and <15% of BIC+FTC+TAF, DTG+FTC+TAF, and DTG+RPV cultures had VB. Emergent reverse transcriptase (RT) or integrase (IN) resistance was seen with DTG+RPV and DTG+3TC but not with BIC+FTC+TAF or DTG+FTC+TAF. At *C*_min_−4, 100% VB occurred with DTG+3TC and DTG+FTC+TAF by day 12, while 94% VB occurred with DTG+RPV by day 25 and only 50% VB occurred with BIC+FTC+TAF by day 35. Emergent *C*_min_−4 drug resistance was seen with all regimens but at differing frequencies; DTG+RPV had the most cultures with resistance. Emergent resistance was consistent with clinical observations. Overall, under high adherence conditions, no *in vitro* VB or resistance development occurred with these INSTI-based regimens. However, when multiple missed doses were simulated *in vitro*, BIC+FTC+TAF had the highest forgiveness and barrier to resistance of all tested regimens. Compared to DTG+3TC and DTG+FTC+TAF, DTG+RPV had higher forgiveness but lower resistance barrier after several simulated missed doses.

## INTRODUCTION

Antiretroviral therapy (ART) for treatment of HIV infection has significantly reduced morbidity and mortality for people living with HIV. However, ART use is lifelong, and there is a risk of treatment interruption or lapses in adherence over the course of taking these medications. Poor adherence is associated with loss of viral control and may lead to the development of drug resistance to one or more drugs in a patient’s regimen (1). “Forgiveness” refers to the ability to achieve or maintain viral suppression despite imperfect adherence to ART. This effect is regimen specific and depends on host, viral, and pharmacological factors (2).

Guidelines for initial treatment of HIV-1 infection primarily recommend three-drug combination ART that includes an integrase strand transfer inhibitor (INSTI) plus 2 nucleos(t)ide reverse transcriptase inhibitors (NRTIs). There are various exclusionary criteria when using different regimens, including preexisting resistance, level of viremia, and coinfection with hepatitis B virus, among others (3–6). Recommended regimens include the single-tablet regimen (STR) bictegravir/emtricitabine/tenofovir alafenamide (BIC/FTC/TAF) and the regimens of dolutegravir (DTG) plus FTC/TAF (DTG+FTC/TAF) and dolutegravir/abacavir/lamivudine (DTG/ABC/3TC). More recently, two-drug combination ART has been explored, and some regimens have shown noninferiority compared to three-drug regimens in carefully selected populations (7–9). The INSTI plus 1 NRTI combination of DTG/lamivudine (3TC) is now included in the guidelines for a subset of patients, and the INSTI plus nonnucleoside reverse transcriptase inhibitor (NNRTI) combination of DTG/rilpivirine (RPV) is approved for patients switching ART regimens. In clinical trials, BIC/FTC/TAF, DTG/ABC/3TC, DTG+FTC/TAF, DTG/3TC, and DTG/RPV have all shown durable efficacy through at least 144 weeks in appropriately selected treatment-naive or virologically suppressed switch participants (10–13). However, infrequent cases of treatment failure and emergent drug resistance have been reported, mostly in people living with HIV (PLWH) with poor adherence and/or advanced HIV disease (12, 14–21). Given the long-term or lifelong nature of ART, it is important to understand the factors that may prevent virologic failure or resistance development.

Forgiveness can be used as a comparative descriptor of different antiretroviral (ARV) regimens; for example, it is known that boosted protease inhibitors (PIs) or NNRTIs are more forgiving of suboptimal adherence than unboosted PIs (22–24). Our group has developed an *in vitro* fixed-dose viral resistance breakthrough (VB) assay that uses clinically relevant drug concentrations in HIV-1-infected cells to evaluate regimen forgiveness and barrier to resistance (25, 26). Using clinical pharmacology information, we can simulate drug exposures at full adherence or suboptimal adherence to treatment *in vitro*. Previous work with this assay system has shown that the three-drug combination BIC+FTC+TAF has more forgiveness and a higher barrier to resistance than the two-drug combination DTG+3TC, demonstrated by less viral breakthrough and less drug resistance development. Here, we evaluated four ARV combinations in parallel, BIC+FTC+TAF, DTG+FTC+TAF, DTG+3TC, and DTG+RPV, to understand the relative time to *in vitro* viral breakthrough and resistance development in our experimental assay system.

## RESULTS

### Determination of cell culture equivalent physiologically relevant drug concentrations.

The pharmacokinetics of the approved INSTI-containing drug regimens BIC/FTC/TAF, DTG+FTC/TAF, DTG/3TC, and DTG/RPV have been studied in clinical trials. To translate *in vivo* drug concentrations to *in vitro* cell culture, clinical pharmacokinetic data were used and adjusted for human plasma protein binding where appropriate. For the INSTIs BIC and DTG and the NNRTI RPV, the median plasma drug concentrations at *C*_min_ from participants in clinical trials are 2.61 μg/mL for BIC, 1.11 μg/mL for DTG, and 0.08 μg/mL for RPV (5,808 nM, 2,515 nM, and 218 nM, respectively) (27–29) (Table 1). These three drugs are highly protein bound but to different extents, and as such only a small fraction of the measured plasma drug concentration is free to enter target cells for inhibition of viral replication. An equilibrium dialysis assay was used to measure the fold change in drug concentration in human plasma and cell culture medium with 10% fetal bovine serum (FBS), and the resulting protein binding factors (PBF; human serum shifts) are 43.6 for BIC, 27.5 for DTG (30), and 32 for RPV (31) (internal data). The cell culture equivalent (CCE) *C*_min_, or the drug concentration in cell culture medium that represents the amount of drug in plasma free to enter target cells, is the clinical *C*_min_ divided by the PBF and is 133 nM for BIC, 91 nM for DTG, and 6.8 nM for RPV. To determine drug concentrations simulating missed doses of daily ARV regimens, the following equation was used: *C*_min_−*X* = *C*_min_ × (0.5^[24×^*^X^*^/^*^t^*^1/2]^); the pharmacologic *in vivo* half-lives for BIC, DTG, and RPV are 17.3 h, 14 h, and 50 h, respectively (27–29).

**TABLE 1:** Cell culture drug concentrations simulating *C*_min_ and *C*_min_ after missing 1 to 4 consecutive doses

Parameter	Value by antiretroviral drug					
	BIC	FTC	TAF	DTG	3TC	RPV
Clinical dose^*a*^ (mg)	50	200	25	50	300	25
Mol wt (g/mol)	449.4	247.2	534.5	419.4	229.3	366.4
Clinical *C*_min_ (μg/mL)	2.61	0.096	0.008	1.11	0.042	0.08
Clinical *C*_min_ (nM)	5808	388	15	2515	265	218
Human serum shift^*b*^	43.6	1.0	1.0	27.5	1.0	32
*t*_1/2_^*c*^ (h)	17	37	116	14	17.5	50
CCE^*d*^ *C*_min_, nM	133	388	15	91	265	6.8
CCE *C*_min_−1, nM	50	248	13	28	102	4.9
CCE *C*_min_−2, nM	19	158	11	8.5	40	3.5
CCE *C*_min_−3, nM	7.1	101	9.8	2.6	15	2.5
CCE *C*_min_−4, nM	2.7	64.2	8.5	0.8	5.9	1.8

a Clinical doses of BIC, FTC, and TAF in the single-tablet regimen of bictegravir/emtricitabine/tenofovir alafenamide and DTG, 3TC, and RPV in the single-tablet regimens of dolutegravir/lamivudine and dolutegravir/rilpivirine (27–29).

b BIC and DTG data generated by standard equilibrium dialysis shift in human serum versus complete cell culture media (30). RPV data were generated internally and are comparable to reported serum shift (31).

c Drug *t*_1/2_ for BIC, DTG, FTC-TP, TFV-DP, 3TC-TP, and RPV (27–29, 35–37).

d Cell culture equivalent (CCE) dose is the clinical *C*_min_/human serum shift ratio; *C*_min_−*X* doses determined as *C*_min_ × (0.5^[24 ×^ *^X^*^/^*^t^*^1/2]^).

To simulate *in vivo* drug concentrations *in vitro* for NRTIs, similar careful calculations were used. Since NRTIs have low protein binding, no protein binding adjustment is required for TAF, FTC, or 3TC. TAF is a prodrug of tenofovir that loads CD4^+^ T cells and is converted to the active metabolite tenofovir diphosphate (TFV-DP) (32). TFV-DP is highly charged and retained in cells with a relatively long intracellular half-life of 116 h (27, 33). *In vitro* experiments have determined the concentration of TFV-DP at *C*_min_ in peripheral blood mononuclear cells (PBMCs) can be reached in cell culture by using 15 nM TAF (34). The active metabolite of FTC is FTC-triphosphate (FTC-TP), with an intracellular half-life of 37 h, and the active metabolite of 3TC is 3TC-triphosphate (3TC-TP), with an intracellular half-life of 17.5 h (35–37). The *in vivo C*_min_ concentrations of FTC and 3TC are 96 nM and 42 nM, respectively, which result in 388 nM FTC-TP and 265 nM 3TC-TP (27–29).

### Viral breakthrough of HIV-1 and resistance development with BIC+FTC+TAF, DTG+FTC+TAF, DTG+3TC, and DTG+RPV.

**FIG 1:**
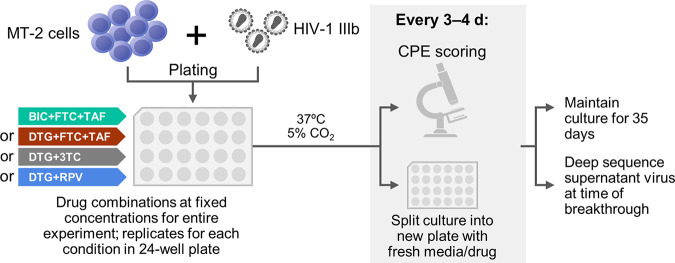
*In vitro* viral breakthrough selections. MT-2 cells were bulk infected with HIV-1 IIIb strain and cultured in replicate on 24-well plates in the presence of fixed concentrations (*C*_min_, C_min_−1, *C*_min_−2, *C*_min_−3, or *C*_min_−4) of BIC+FTC+TAF, DTG+FTC+TAF, DTG+3TC, or DTG+RPV. Infected cultures were split every 3 to 4 days with fresh medium containing drugs and closely monitored for viral breakthrough by cytopathic effect (CPE) for up to 35 days of selection. Cell-free supernatants containing breakthrough virus were collected upon emergence and stored frozen for deep sequencing.

**FIG 2:**
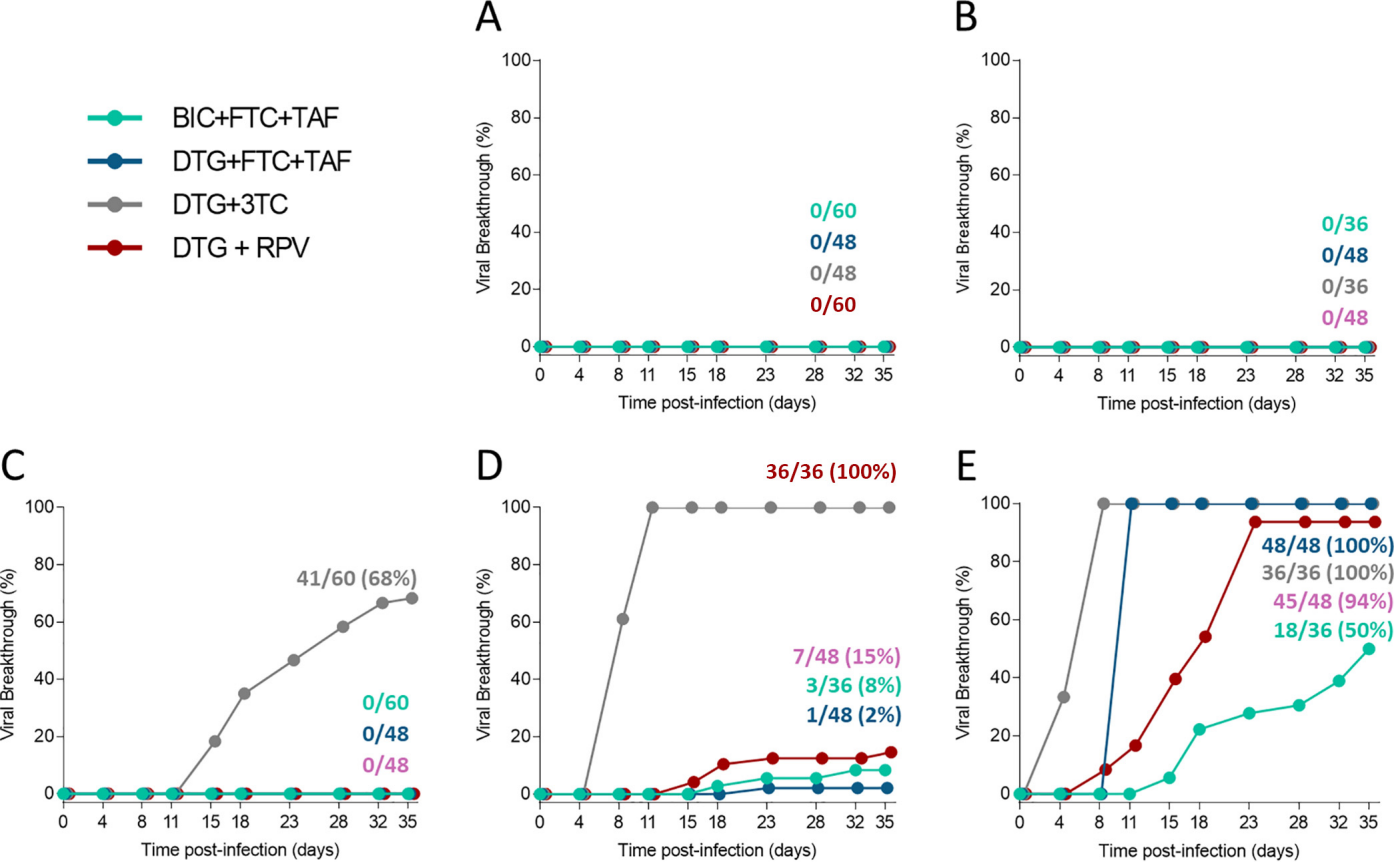
Time to viral breakthrough. Time to viral breakthrough in MT-2 cells infected with wild-type HIV-1 IIIB strain is shown. Viral breakthrough selections for each drug combination were tested in replicate infected cultures in the presence of constant drug pressure for up to 35 days or until viral breakthrough was observed. The number of cultures with viral breakthrough by observed cytopathic effect was scored at each time point. Selections were performed at the following drug concentrations: simulated *C*_min_, the minimum drug exposures based on *in vivo* pharmacokinetics (A); simulated *C*_min_ after missing 1 dose (*C*_min_−1) (B); *C*_min_ after missing 2 consecutive doses (*C*_min_−2) (C); *C*_min_ after missing 3 consecutive doses (*C*_min_−3) (D); and *C*_min_ after missing 4 consecutive doses (*C*_min_−4) (E). Symbols have been slightly offset on the *x* axis to aid viewing.

Viral breakthrough assays using the drug combinations of BIC+FTC+TAF, DTG+FTC+TAF, DTG+3TC, and DTG+RPV were performed in parallel at fixed drug concentrations simulating *C*_min_, *C*_min_ minus one missed daily dose (*C*_min_−1), and *C*_min_−2, *C*_min_−3, and *C*_min_−4 consecutive missed daily doses. Briefly, MT-2 cells were infected with HIV-1 IIIB wild-type virus and maintained at single fixed drug concentrations in replicate cell cultures for up to 35 days; cultures were split every 3 to 4 days, and supernatant was harvested when viral breakthrough was observed by widespread cytopathic effect in the cell culture (Fig. 1). For each of the four drug combinations tested (BIC+FTC+TAF, DTG+FTC+TAF, DTG+3TC, and DTG+RPV), no viral breakthrough occurred at drug concentrations corresponding to full adherence and one missed dose (*C*_min_ and *C*_min_−1) (Fig. 2A and B). At cell culture equivalent trough drug concentrations corresponding to two consecutive missed doses (*C*_min_−2), there was no viral breakthrough for BIC+FTC+TAF, DTG+FTC+TAF, or DTG+RPV (Fig. 2C). DTG+3TC had viral breakthrough starting at day 14 and had 41/60 cultures break through by the end of study (day 35). In these experiments, the supernatant virus was genotyped by next-generation sequencing, and resistance mutations present at ≥2% frequency were reported. In total, 13 of the DTG+3TC breakthrough cultures at the *C*_min_−2 drug concentration had emergent reverse transcriptase (RT) and/or integrase (IN) substitutions associated with drug resistance (Table 2). The most frequent substitution was M184V/I in RT in 4 cultures, present at 2.2% to 42.2% prevalence. This mutation confers high-level resistance to 3TC and FTC and hypersusceptibility to TAF and is the most frequent resistance mutation that emerges at virologic failure in PLWH treated with NRTIs (38–40). The S153F variant in IN was selected in 1 culture, and this mutation confers low-level reduced susceptibility to DTG and BIC and has been selected in PLWH and in resistance selections *in vitro* (30, 41, 42). Other drug-associated mutations selected were V75I in RT and L74M, G140E/R, or E157K in IN. These single mutations show no or minimal phenotypic resistance to 3TC or DTG but may increase drug resistance or viral fitness when combined with primary drug resistance mutations (41). The observation of these drug-associated mutations at lower levels by next-generation sequencing suggests that the virus is evolving to escape drug pressure. Evolution of patterns of resistance has been seen in patients; therefore, these *in vitro* developed mutations may further evolve to show phenotypic resistance (43, 44).

**TABLE 2:** Resistance

*In vitro* drug concentration	Breakthrough frequency (resistance development)							
	BIC+FTC+TAF		DTG+FTC+TAF		DTG+3TC		DTG+RPV	
	VB (*n/N*; %) [first day of VB]	With resistance, N^*a*^	VB (*n/N*; %) [first day of VB]^*b*^	With resistance, N^*a*^	VB (*n/N*; %) [first day of VB]	With resistance, N^*a*^	VB (*n/N*; %) [first day of VB]	With resistance, N^*a*^
*C*_min_	0/60; 0 [NA]	0	0/48; 0 [NA]	0	0/60; 0 [NA]	0	0/48; 0 [NA]	0
*C*_min_−1	0/36; 0 [NA]	0	0/48; 0 [NA]	0	0/36; 0 [NA]	0	0/48; 0 [NA]	0
*C*_min_−2	0/60; 0 [NA]	0	0/48; 0 [NA]	0	41/60; 68 [14]	13; RT, M184V/I (4), V75I (3); *IN*, *G140E/R* (2), *E157K* (2), *L74M* (1), *S153F* (1)	0/48; 0 [NA]	0
*C*_min_−3	3/36; 8 [21]	0	1/48; 2 [25]	0	36/36; 100 [7]	3; RT, none; *IN*, *L74M* (2), *V72A* (1), *S153F* (1)	7/48; 15 [14]	1; RT, M230I; *IN*, none
*C*_min_−4	18/36; 50 [15]	3; RT, M184I (2); *IN*, *G163R* (1)	48/48; 100 [11]	6; RT, M184V (1), K219R (1); *IN*, *Q148R* (2), *Q95R* (1), *H51Y* (1), *S153F* (1)	36/36; 100 [5]	2; RT, none; *IN*, *R263K* (2), *L74M* (1)	45/48; 94 [8]	20; RT, E138K (8), K101E (3), M230I (2), V90I (2), V106I (1), Y181C (1), H221Y (1); *IN*, H51Y (2), *R263K* (1), *M50I* (1), *Q95R* (1), *A128T* (1), *S153F* (1), *G163R* (1)

a Reverse transcriptase (RT) substitutions are shown in plain text. Integrase (IN) substitutions are shown in italics. Some viral breakthrough supernatants had more than one emergent resistance mutation, as listed here: DTG+3TC *C*_min_−3, 1 *L74M+S153F* in IN; DTG+3TC *C*_min_−4, 1 *L74M+R263K* in IN; DTG+FTC+TAF *C*_min_−4, 1 M184V in RT + *Q95R* in IN; DTG+RPV *C*_min_−4, 1 V90I+V106I+E138K in RT, 1 Y181C in RT + *H51Y* in IN, 1 E138K in RT + *H51Y* in IN, 1 E138K in RT + *Q95R* in IN, and 1 E138K in RT + *A128T* in IN.

b VB, viral breakthrough; NA, not applicable.

At *C*_min_−3 drug concentrations, viral breakthrough occurred for all four drug combinations tested (BIC+FTC+TAF, DTG+FTC+TAF, DTG+3TC, and DTG+RPV) but differed by time and extent of breakthrough (Fig. 2D). DTG+3TC had viral breakthrough first starting at day 7, with all cultures (36/36) breaking through by day 12 and IN resistance mutations in 3 of these cultures. BIC+FTC+TAF, DTG+FTC+TAF, and DTG+RPV had fewer cultures with breakthrough that were detected later than that for DTG+3TC. DTG+RPV breakthrough was first detected at day 14 and reached 7/48 cultures through day 35; BIC+FTC+TAF breakthrough was detected at day 21 and reached a maximum of 3/36 cultures; DTG+FTC+TAF had 1/48 cultures with breakthrough at day 25. The NNRTI resistance mutation M230I was detected in 1 DTG+RPV breakthrough culture, and no resistance was detected in the BIC+FTC+TAF or DTG+FTC+TAF breakthrough cultures.

At *C*_min_−4 drug concentrations for all regimens tested, viral breakthrough occurred earlier and to a greater extent than at higher drug concentrations (Fig. 2E). All DTG+3TC and DTG+FTC+TAF cultures (36/36 and 48/48, respectively) showed viral breakthrough by day 12. Two of the DTG+3TC cultures with breakthrough developed IN resistance, both with the R263K mutation. R263K confers about 2-fold resistance to both DTG and BIC and has been documented in rare cases of virologic failure (20, 42, 45–48). Six of the DTG+FTC+TAF breakthrough cultures developed RT or IN resistance, including 1 with M184V in RT and 2 with Q148R in IN; these mutations have been selected in patients receiving DTG-based therapy (49–51). The DTG+RPV selections had 94% (45/48 cultures) breakthrough by day 25. Twenty of the DTG+RPV breakthrough cultures developed resistance to RT or IN, with some cultures developing multiple mutations in one or both genes. Resistance mutations included the major RPV-associated mutations E138K, Y181C, and K101E as well as the IN mutations R263K and S153F. BIC+FTC+TAF was the combination associated with the slowest breakthrough, which was first detected at day 15 and reached 50% (18/36 cultures) by day 35. Three of the BIC+FTC+TAF breakthrough cultures developed resistance to components of the regimen; the mutations observed were the RT mutation M184I in 2 cultures and the IN mutation G163R in 1 culture. Overall, these four INSTI-containing regimens had no viral breakthrough at *C*_min_ drug concentrations, as expected, but as multiple missed doses were simulated, the regimens had breakthrough and resistance development to different extents.

## DISCUSSION

Major factors that lead to virologic failure and emergent drug resistance are high baseline viral loads, low baseline CD4^+^ T cell counts, and poor adherence to ART (52). Intermittent adherence using a structured treatment interruption strategy was attempted to decrease the exposure to HIV drugs with substantial toxicities; these interventions of antiretroviral regimens resulted in virologic rebound and resistance development and are not recommended in routine clinical care (53, 54). Some studies of less frequent dosing of currently recommended daily oral regimens are being conducted and may yield useful information on the risks to people with suboptimal adherence; however, these alternative dosing strategies should be undertaken with caution. Since clinical trials studying suboptimal adherence and potentially virologic failure could be risky for study participants, we have conducted studies simulating imperfect adherence and forgiveness of four treatment regimens *in vitro*.

This study evaluated the four regimens BIC/FTC/TAF, DTG+FTC/TAF, DTG/3TC, and DTG/RPV in viral breakthrough selection experiments to understand the relative time to *in vitro* viral breakthrough and resistance development. For each of the four ART regimens tested here, no viral breakthrough occurred at drug concentrations corresponding to full adherence or to one missed dose (*C*_min_ and *C*_min_−1). This would be expected based on pharmacokinetic and virologic failure data from clinical trials. When drug concentrations were evaluated that corresponded to having missed two consecutive doses (*C*_min_−2), DTG+3TC was the only regimen that allowed viral breakthrough, with emergence of resistance mutations to RT or IN in some cultures. At *C*_min_−3 and *C*_min_−4 drug concentrations, all regimens allowed viral breakthrough; however, DTG+3TC consistently had the earliest and most extensive breakthrough. Interestingly, at *C*_min_−4, breakthrough occurred in 100% of DTG+FTC+TAF cultures, beginning at day 11. In contrast, breakthrough occurred in only 50% of BIC+FTC+TAF cultures, with the first breakthrough at day 15. This observation was seen with multiple independent experiments. The BIC/FTC/TAF and DTG+FTC/TAF regimens both demonstrate high efficacy in clinical trials, where frequent clinic visits and adherence counseling lead to higher adherence than in the real world (10, 55). However, there are pharmacokinetic, pharmacodynamic, and resistance differences between BIC and DTG that may have contributed to the difference seen in this study. BIC achieves higher drug exposures than DTG, has a longer effective plasma half-life, has more contacts with the IN-DNA target, has a longer dissociation half-life from the IN-DNA complexes, and has a more favorable resistance profile than DTG (30, 47, 56–59). At simulated *C*_min_−4, DTG+RPV broke through more slowly than DTG+3TC and DTG+FTC+TAF and did not achieve complete viral breakthrough by day 35 (end of selection experiment); however, these DTG+RPV breakthrough cultures developed more resistance than any of the other regimens. Cumulatively across all regimen conditions tested in this series, emergent RT and/or IN resistance was detected in 3/228 (1.3%) of BIC+FTC+TAF, 6/240 (2.5%) of DTG+FTC+TAF, 18/228 (7.9%) of DTG+3TC, and 21/240 (8.8%) of DTG+RPV breakthrough cultures. Both 2-drug combinations had more overall resistance development than the 3-drug combinations; this could be due to higher cumulative drug concentrations, more combinations with antiviral synergy, better activity against resistance mutations that emerge, and hypersusceptibility of the M184V resistance mutant to TAF.

The viral breakthrough trends observed with the four regimens studied here correlate with published clinical data. Overall, all the regimens have demonstrated high efficacy in clinical trials, and there have been no cases of virologic failure with resistance for participants taking BIC/FTC/TAF or DTG+FTC/TAF and few with DTG/3TC or DTG/RPV (7, 10, 12–15, 60–62). However, it is important to recognize that adherence in clinical trials is higher than that in real-world situations (63), and some of these clinical trials had limitations on baseline viral load, baseline resistance to study drugs, and/or hepatitis B virus coinfection. In one study looking at the impact of adherence on viral suppression with BIC- and DTG-containing triple therapy regimens, it was found that good adherence, above thresholds of 80% or 95%, independently predicted viral suppression at 6 months with either DTG/ABC/3TC or DTG multitablet combinations (DTG+FTC/TAF, DTG+FTC/TDF, and DTG+ABC+3TC) but not with BIC/FTC/TAF (64). In other words, better adherence resulted in greater viral suppression with the DTG-based regimens, but response to BIC/FTC/TAF did not depend on adherence. There are limited data on adherence and forgiveness with the two-drug regimens DTG/3TC or DTG/RPV, but the data cited here do suggest that BIC/FTC/TAF has higher forgiveness than other prescribed INSTI-containing regimens.

The resistance mutations observed in this study were also consistent with resistance observed clinically. The major mutations that emerge in PLWH with virologic failure are M184V/I in RT for FTC or 3TC, R263K for DTG or BIC or Q148R in IN for DTG, and K101E, E138K, Y181C, M230I, or H221Y in RT for RPV. Although development of resistance is low for all of these regimens, drug resistance can have severe consequences, and there have been reported cases of resistance in people that are consistent with those from our *in vitro* system. In clinical trials enrolling treatment-naive participants, noncoformulated DTG+3TC has selected for the M184V mutation in RT plus the R263K mutation in IN in 2 cases, both involving nonadherent trial participants (12, 14). DTG+RPV has selected for K101E, E138E/A, and M230L in RT in clinical trials (13, 65, 66) and E138Q and Y181C in RT in clinical practice (67). Resistance has been reported in treatment-naive individuals taking DTG+FTC+TDF in clinical practice with the mutations M184V/I in RT and G118R, E157Q, Q148K, and R263K in IN (68–71). In these cases, risk factors for resistance included advanced disease, comorbidities, high baseline viral load, and/or low baseline CD4 cell count and poor adherence. Rare cases of emergent resistance in clinical practice on BIC+FTC+TAF have seen emergent M184V/I in RT and H51Y, E138K, S147G, and R263K in IN (17–19). In these cases, risk factors for resistance included advanced disease, prior virologic failure on an INSTI-containing regimen, and/or poor adherence.

These *in vitro* models have limitations. There is not a direct translation of simulated missed doses *in vitro* to *in vivo* missed doses; this *in vitro* system is likely more sensitive to missed doses than *in vivo*, where the immune system would contribute to viral suppression and help keep HIV-1 in a latent state (72). *In vivo*, reactivation of virus may begin in the lymph node and take time to progress to detectable plasma viremia versus rapid spread and detection in culture. In addition, drug distribution in the body is heterogeneous and ARV penetration in tissues can be poor (73). Although we studied consecutive missed doses of drug here, suboptimal adherence can take many forms, including other patterns of missing doses of drug, administration with contraindicated medications that may decrease exposure, or insufficient food requirements. It is also important to recognize a major reason for poor adherence are side effects in PLWH, and although a drug combination may have high forgiveness or a high resistance barrier in this assay, the combination may have more side effects, which could lead to poor clinical adherence. In addition, drug concentrations here were kept constant for experimental consistency, whereas *in vivo*, drug concentrations reach a maximum concentration and then continually decline. For the 3TC and FTC drug concentrations, the cell culture clinical *C*_min_ is below the 50% effective concentration (EC_50_) of these drugs in this system; however, synergy between drugs and hypersusceptibility of TAF to the M184V virus may have prevented some resistance development. Still, this model is one way to study missed doses and viral breakthrough *in vitro* in a controlled environment and may highlight differences between regimen potency under conditions simulating short periods of nonadherence.

Overall, there was no viral breakthrough or resistance development with these INSTI-based combinations under high-adherence conditions. When multiple missed doses were simulated *in vitro*, the drug combinations had different levels of forgiveness and barriers to resistance. BIC+FTC+TAF had the highest forgiveness and barrier to resistance. DTG+RPV had higher forgiveness but lower resistance barrier after multiple missed doses compared to DTG+3TC and DTG+FTC+TAF. These data suggest that higher drug levels, distinct resistance profiles, and antiviral synergy are more protective in individuals with suboptimal adherence and should be considered when choosing ARV regimens, particularly in the real world, where imperfect drug adherence is expected.

## MATERIALS AND METHODS

### Reagents, cell culture, and HIV strains.

BIC, FTC, 3TC, TAF, and RPV were synthesized at Gilead Sciences, Inc. (Foster City, CA, USA). DTG was purchased from Porton Pharma Solutions (Shanghai, China). All drug stocks were prepared in 100% dimethyl sulfoxide (DMSO), and potency in tissue culture was consistent with the literature values. The HTLV-1-transformed human T cell line MT-2 was obtained from the NIH AIDS Reagent Program (Germantown, MD, USA) (74, 75) and maintained at 37°C in 5% CO_2_ at densities below 1 × 10^6^ cells/mL by serial passaging in RPMI cell culture medium supplemented with 10% heat-inactivated fetal bovine serum (FBS) and antibiotics (Sigma-Aldrich, St. Louis, MO, USA). The laboratory-adapted HIV-1 IIIB strain (NIH AIDS Reagent Program) was used for infection (76).

### Drug concentration determination.

The clinical minimum drug concentration (*C*_min_), defined as the steady-state trough plasma drug concentration, was obtained from individual drug package inserts for BIC/FTC/TAF (Biktarvy), DTG/3TC (Dovato), and DTG/RPV (Juluca) (27–29). A previously described standard equilibrium dialysis assay was used to directly measure the differences between free drugs in human plasma and cell culture medium (77). Briefly, 100% human plasma containing drug was added to one dialysis chamber, and cell culture medium (CCM; supplemented with 10% FBS) containing the same amount of drug was added to the second dialysis chamber. The chambers were then rotated for 3 h in a 37°C water bath, after which time the drug concentration in each chamber was determined by liquid chromatography-mass spectrometry (LC-MS). The resulting ratio, representing the fold difference in drug concentration in human plasma and CCM, identified the human plasma shift value for each drug. This shift was used to generate the cell culture equivalent (CCE) *C*_min_ drug concentration (clinical *C*_min_/human plasma shift) that was used for BIC, DTG, and RPV. Determination of TAF *C*_min_ concentration was described previously and generated by correlating intracellular tenofovir-diphosphate (TFV-DP) with its physiological concentration in peripheral blood mononuclear cells (PBMCs) from TAF-treated individuals (34). FTC and 3TC concentrations were not adjusted for protein binding and were set at their human plasma *C*_min_ concentrations (35, 36). To simulate one, two, three, or four consecutive missed doses (*C*_min_−1, *C*_min_−2, *C*_min_−3, and *C*_min_−4), drug concentrations were adjusted by their plasma half-lives for BIC, DTG, and RPV and active metabolite half-lives for the NRTIs (TAF, FTC, and 3TC). *C*_min_−*X* doses was determined as *C*_min_ × (0.5^[24 ×^ *^X^*^/^*^t^*^1/2]^) (27–29, 35, 36).

### HIV-1 breakthrough selections in MT-2 cells.

MT-2 cells were infected with HIV-1 IIIB at a multiplicity of infection (MOI) of 0.05 for 3 h and plated in 24-well plates at 2 × 10^5^ cells per well (Fig. 1). Experiments with each drug combination studied *C*_min_, C_min_−1, *C*_min_−2, *C*_min_−3, and *C*_min_−4 at fixed drug concentrations over the course of the experiment. Drugs were added 16 h after infection to a minimum of 12 replicate cultures at fixed concentrations equal to their CCE *C*_min_ concentration or at concentrations adjusted for missing doses at 24, 48, 72, and 96 h (1, 2, 3, and 4 doses, respectively). Every 3 to 4 days, cells were diluted (1:5) into freshly prepared drug media and monitored for virus-induced cytopathic effects (CPE) over a period of 35 days. Cell-free viral supernatants were harvested from cultures showing >90% CPE and kept frozen at −80°C until further analyses.

### Sequencing of breakthrough HIV-1 variants.

A next-generation sequencing/deep sequencing analysis of HIV-1 protease, RT, and IN was conducted on viral breakthrough samples. Sequencing used the DeepType HIV assay (Seq-IT) and Gilead-developed software to process and align data and identify substitutions present (78, 79). Resistance-associated substitutions were analyzed at a frequency cutoff of ≥2% prevalence.

### Data availability.

Sequencing data related to this study have been deposited in NCBI under BioProject no. PRJNA812639.
